# Combining Unsupervised and Supervised Learning for Sample Efficient Continuous Language Grounding

**DOI:** 10.3389/frobt.2022.701250

**Published:** 2022-09-30

**Authors:** Oliver Roesler

**Affiliations:** Artificial Intelligence Lab, Vrije Universiteit Brussel, Brussels, Belgium

**Keywords:** language grounding, cross-situational learning, interactive learning, sample efficiency, human-agent interaction, CLEVR

## Abstract

Natural and efficient communication with humans requires artificial agents that are able to understand the meaning of natural language. However, understanding natural language is non-trivial and requires proper grounding mechanisms to create links between words and corresponding perceptual information. Since the introduction of the “Symbol Grounding Problem” in 1990, many different grounding approaches have been proposed that either employed supervised or unsupervised learning mechanisms. The latter have the advantage that no other agent is required to learn the correct groundings, while the former are often more sample-efficient and accurate but require the support of another agent, like a human or another artificial agent. Although combining both paradigms seems natural, it has not achieved much attention. Therefore, this paper proposes a hybrid grounding framework which combines both learning paradigms so that it is able to utilize support from a tutor, if available, while it can still learn when no support is provided. Additionally, the framework has been designed to learn in a continuous and open-ended manner so that no explicit training phase is required. The proposed framework is evaluated through two different grounding scenarios and its unsupervised grounding component is compared to a state-of-the-art unsupervised Bayesian grounding framework, while the benefit of combining both paradigms is evaluated through the analysis of different feedback rates. The obtained results show that the employed unsupervised grounding mechanism outperforms the baseline in terms of accuracy, transparency, and deployability and that combining both paradigms increases both the sample-efficiency as well as the accuracy of purely unsupervised grounding, while it ensures that the framework is still able to learn the correct mappings, when no supervision is available.

## 1 Introduction

The most natural form of communication between humans is natural language, which allows a person to transmit knowledge to another person or to request another person to perform a specific action Ellis (1993). Enabling artificial agents to become accepted partners and collaborate with humans in a natural and efficient way therefore requires the artificial agents to understand natural language. However, understanding natural language is non-trivial and requires agents to ground natural language in the real world by creating connections between symbols, i.e., words or phrases, and their meanings, i.e., perceptual information extracted through the agents’ sensors from the environment. A variety of grounding approaches have been proposed in the literature, which either utilize supervised or unsupervised learning techniques to obtain links between words and corresponding concrete representations^1^. The latter represent sets of invariant perceptual features obtained through an agent’s sensors that are sufficient to distinguish percepts belonging to different concepts^2^. Supervised approaches are usually sample efficient because the employed tutors, which can be both humans or other artificial agents, actively support the grounding process and either prevent the creation of wrong mappings or ensure that they are quickly removed, however, these approaches depend on the availability and trustworthiness of a tutor and fail, if no supervision is available or the tutor provides false information. An example is the Grounded Naming Game (Steels and Loetzsch, 2012), which is an interactive learning based approach that has been applied in different studies to ground colors, spatial relations and other modalities (Bleys et al., 2009; Spranger, 2013). In contrast to the previously described approaches, unsupervised approaches avoid this dependency on supervision and utilize instead co-occurrence information, i.e., how often a specific symbol appears together with a specific concrete representation. The advantage is that they work without the support of a tutor, however, they are less sample efficient and often also less accurate. Examples are cross-situational learning (Siskind, 1996; Smith et al., 2011) based approaches that have been used to ground objects, actions, and spatial concepts (Dawson et al., 2013; Aly et al., 2017). Only limited work, i.e., (Belpaeme and Morse, 2012; Nevens and Spranger, 2017; Roesler, 2020a), has been done to compare or combine both approaches (see Section 2.3). Therefore, this study proposes and evaluates a hybrid grounding framework that combines both paradigms. More specifically, this study extends a recently proposed unsupervised cross-situational learning based grounding framework (Roesler, 2020b), which has been shown to achieve state-of-the-art grounding results, with a novel interactive learning based mechanism to learn from feedback provided by a tutor. The hypothesis is that the hybrid framework is more sample efficient and produces more accurate groundings than frameworks that use only unsupervised learning, while at the same time being able to work in the absence of supervision, which is not the case for purely supervised frameworks. Therefore, the main research questions investigated in this study are: 1) Do the proposed feedback mechanisms increase the sample efficiency of the unsupervised grounding framework and the accuracy of the obtained groundings? 2) Does combined verbal and pointing feedback^3^ has a stronger positive influence on the grounding performance than pointing-only feedback? 3) Does the model perform as well as state-of-the-art unsupervised grounding models, if no supervision is provided. To verify the hypothesis and investigate above research questions, two different human-agent interaction scenarios are employed, which require the agent to ground synonymous shape, color, action, and preposition words through geometric characteristics of objects, color mean values, action feature vectors, and spatial vectors. Additionally, grounding results are obtained for different feedback rates, i.e., for different amounts of supervision, to investigate whether more feedback leads to better groundings.The remainder of this paper is structured as follows: Section 2 provides an overview of related work. The proposed hybrid grounding framework and the employed experimental setup are described in Sections 3 and 4. The obtained grounding results are presented and evaluated in Section 5. Finally, Section 6 concludes the paper.

## 2 Related Work

Since this paper investigates the combination of unsupervised and supervised grounding approaches for language grounding, this section describes related work employing purely unsupervised or supervised mechanisms as well as the limited number of works that have, at least partially, addressed the combination of both paradigms.

### 2.1 Unsupervised Grounding

The motivation for unsupervised grounding approaches comes from the fact that children are able to learn the meaning of words, i.e. ground them in the real world, without any explicit teaching or supervision by already proficient language users, e.g., their parents or other adults (Bloom, 2001). One possible mechanism that allows children to ground words in an unsupervised manner and without the need for a tutor is cross-situational learning, which allows to learn the meaning of words across multiple exposures while handling referential uncertainty. The main idea of cross-situational learning is that a set of candidate meanings, i.e., mappings from words or phrases to corresponding concrete representations, can be created for every situation a word is used in and that the correct meaning is located where the sets of candidate meanings intersect so that the correct word-concrete representation mappings will reliably reoccur across situations (Pinker, 1989; Fisher et al., 1994; Blythe et al., 2010; Smith and Smith, 2012). Several experimental studies have confirmed that humans employ cross-situational learning for word learning, if no prior knowledge of language is available. For example, Akhtar and Montague (1999) conducted a study with 24 two-, three- and four-year-olds in which the children were presented with novel objects that differed in their shape and texture. During the experiment a new artificial adjective was introduced by telling the child “This is a *adjective* one,” where *adjective* referred to the shape or texture of the target object. Afterwards, several other objects were shown to the child that had the same characteristic referred to by the used *adjective*. The results showed that two-year-olds are already able to use cross-situational learning to infer the meaning of initially unknown words. In a different study by Smith and Yu. (2008), 28 12-month-old and 27 14-month-old infants were presented 30 times for four seconds with pictures of two objects on a screen while the name of one of the objects was played via a loudspeaker. During the whole experiment the eye gaze of the infants was recorded to identify for how long they looked at each of the displayed objects and the results showed that they looked longer at the target than the other object, thus, confirming the successful use of cross-situational learning for world learning in infants. Due to the results obtained in the experimental studies with infants and children, a variety of algorithms has been proposed to simulate cross-situational learning in humans and enable artificial agents, such as robots, to learn the meaning of words by grounding them through corresponding concrete representations. Fontanari et al. (2009) applied a Neural Modeling Fields Framework to a grounding scenario in which a tutor presents two objects to a learner while uttering a word that refers to one of the objects so that the learner can infer the correct word-object mapping utilizing co-occurrence information across several situations. While the framework is overall able to infer the correct word-object mappings, it has several drawbacks. First, it requires the data of all situations to be presented at once and is therefore not able to learn in a continuous fashion that is required in realistic scenarios in which unseen words or objects can occur at any time. Second, it is not clear whether the framework can handle real noisy perceptual data because the used concrete representations were perfect and not created from real perceptual data. Finally, the model has only been evaluated for an extremely simple scenario that only contained a single modality and one word utterances without auxiliary words. Tellex et al. (2011) and Dawson et al. (2013) used probabilistic graphical models to ground spatial language through corresponding concrete representations in an offline fashion using large corpora of examples. The employed models performed well for sentences that only contained words they had encountered during training but had problems when sentences contained unknown words. This problem can be addressed through the use of larger datasets, however, they are not easy to obtain because the models require detailed annotations to learn from and it is impossible to create a dataset including all existing words with all possible meanings because language is constantly changing, i.e., new words or meanings are created. Another limitation of the models is that they are not able to handle synonyms, i.e., multiple words referring to the same concept^4^, which is a substantial limitation because many words are synonymous in specific contexts^5^. Aly et al. (2017), Roesler et al. (2018), and Roesler et al. (2019) also employed probabilistic models for grounding, however, they used different experimental setups, grounded different modalities, i.e., spatial relations, actions and shapes, and investigated different research questions. For example, Roesler et al. (2019) investigated the utility of different word representations for grounding of unknown synonyms, which are words for which at least one of their synonyms have been encountered during training while the word itself was not encountered. The results showed that representing words through semantic vectors obtained via Word2Vec^6^ leads to better grounding of unknown synonyms than representing each word through a different symbol, e.g., a number, that encodes no additional information. However, Roesler et al. (2018) showed that for known synonyms representing words through simple symbols leads to better groundings if the semantic information contains noise. Thus, in this study words are represented through simple symbols because in contrast to all studies described above, which required perceptual data and words to be collected in advance for offline training, the employed framework is able to continuously learn new groundings so that all synonyms are known synonyms because no separate offline training phase is necessary. Furthermore, in contrast to the scenario used in this study, none of the scenarios used in the described studies contained homonyms, i.e. one word refers to multiple concepts.

### 2.2 Supervised Grounding

The motivation for supervised grounding approaches is that although children do not need any support to learn their native language, there is evidence that active support by tutors, e.g., their parents or other language proficient people, simplifies word learning and therefore makes children learn faster (Bloom, 2001). Similar to cross-situational learning based approaches, interactive learning based approaches are inspired by studies about how infants and young children learn words. For example, Horst and Samuelson (2010) conducted a study with 24-months old infants investigating whether they could sufficiently learn the names of several novel objects so that they were able to remember them after five minutes, which is a large enough delay to require retrieval from long-term memory. The experiments conducted in the study consisted of two main parts. First, the novel object names were taught by presenting two familiar objects with one novel object. The results showed that the children picked the target object on average more than 70% of the times, independent of whether the experimenter asked for a familiar or novel object. However, when they were presented with two previously novel objects that had been named during the first part of the experiment and one novel object they did not know the name of, they only picked objects requested by the experimenter at chance level when no feedback was provided during the first part of the experiment. In contrast, when feedback was provided in form of extensive labeling, i.e., after the child selected an object the experimenter held up the correct object and pointed to it while stating its name, e.g., “Look, this is the dog!,” the number of times the correct object was selected was around 70%. Thus, feedback in the form of extensive labeling significantly increased the children’s word learning performance. In a different study, Bedford et al. (2013) investigated word learning differences between 31 24-month-old infants at low and high risk for Autism Spectrum Disorder (ASD), which is a neurodevelopmental condition leading to deficits in social communication and interaction (American Psychiatric Association and others, 2013). At the beginning of the experiment the children were introduced to all objects used during the experiment without naming them so that the novelty of objects had no influence on the obtained results. Afterwards, an experimenter showed several objects to the child, while asking to select a specific one, e.g. “Can you give me the moxi?”. Once the child had chosen one of the objects, the experimenter either provided feedback by holding the correct object in front of the child and saying, e.g. “Yes/No, this is the moxi. What a nice moxi!,” or just said “Thank you” without providing any feedback (Bedford et al., 2013). Finally, after the child was allowed to play for 5 minutes with other toys, the experimenter showed the child four times pairs of objects of which only one had been named during the experiment to investigate whether the child remembered which object belonged to the provided name. For two of the four target objects used during this phase, feedback had been provided during the previous phase, while for the other two no feedback had been provided. The results showed that providing feedback increased the number of words the children learned and that this increase was larger for the children that had a lower risk for ASD. Inspired by the previous studies with children, supervised or interactive grounding approaches try to utilize the support of a tutor to obtain word-concrete representation mappings in a sample efficient and highly accurate manner. The main idea is that direct teaching and feedback prevents an artificial agent from learning wrong mappings and reduces the complexity of language grounding by limiting the number of possible mappings. She et al. (2014) used a dialog system to ground higher level symbols through already grounded lower level symbols during human-robot interactions^7^. While the system was able to obtain correct mappings in a fast and interactive manner, the applicability of the proposed system is rather limited because it requires a sufficiently large set of grounded lower level symbols as well as a professional tutor to answer its questions. Especially, the former is difficult to obtain because it is impossible to know in advance what situations an agent will encounter after deployment in the real world and therefore which grounded lower level symbols need to be available. Thus, the presented grounding approach is inadequate as the main or sole grounding mechanisms, while it can be useful in combination with other grounding mechanisms that do not require the existence of already grounding lower level symbols and can therefore be used to obtain them. The need for a human tutor that knows the correct mappings also limits the applicability of the Grounded Naming Game (Steels and Loetzsch, 2012), which has been shown to allow artificial agents to quickly learn word-concrete representation mappings in an interactive game like manner. The used procedure is relatively simple, i.e., an agent gets an instruction, selects the target object by pointing at it, and receives immediate feedback from a human tutor (Bleys et al., 2009; Spranger, 2013). The mechanism works very well because the feedback enables the agent to substantially decrease the set of possible mappings by restricting the set of possible concrete representations a word can be mapped to. Another important constraint used in many studies that employed the Grounded Naming Game methodology is that only a single word or phrase referring to a specific attribute of an object is provided which is completely different from real utterances used by humans that consist of many words^8^. Due to the efficiency and simplicity of the Grounded Naming Game methodology and the fact that it does not require any prior knowledge or previously obtained groundings, the feedback mechanism employed by the proposed hybrid grounding framework follows a similar approach (Section 3.3).

### 2.3 Hybrid Grounding

Combining unsupervised and supervised grounding approaches has so far not received much attention despite the potential to combine their strengths and eliminate or at least reduce the impact of their shortcomings. Nevens and Spranger (2017) investigated the combination of cross-situational and interactive learning and came to the conclusion that the more feedback is provided, the faster new mappings are obtained and the higher the accuracy of the obtained mappings. While these findings, i.e., that feedback improves the accuracy and sample-efficiency, seem reasonable and intuitive, the employed cross-situational learning algorithm was very limited, thus, it is not clear whether feedback would have provided the same benefit, if a more sophisticated unsupervised grounding mechanism would have been employed. A different study by Roesler (2020a) extended an unsupervised cross-situational learning based grounding framework, which has achieved state-of-the-art grounding performance (Roesler, 2020b), with a mechanism to learn from explicit teaching and showed that explicit teaching increases the convergence speed towards the correct groundings. The main disadvantage of the employed supervised learning mechanism is that it requires the tutor to artificially create a special teaching situation, which is a simplified version of the environment specifically designed to ensure that the agent will correctly learn a specific mapping. Finding a tutor who is able and willing to put this amount of effort into teaching the agent, is very unlikely. Since in both studies one of the employed mechanisms, i.e. the unsupervised mechanism in (Nevens and Spranger, 2017) and the supervised mechanism in (Roesler, 2020a), were quiet limited, this study combines two mechanisms that have previously been shown to achieve state-of-the-art grounding results and evaluates whether their combination leads to better sample-efficiency and accuracy, while ensuring that supervision can be provided in a simple and natural way, and is not required to learn the correct groundings.

## 3 Grounding Framework

The proposed *hybrid* grounding framework consists of three main parts: 1) Percepts clustering component (Section 3.1), which determines the corresponding concrete representations for encountered percepts through clustering, 2) Unsupervised grounding component (Section 3.2), which detects auxiliary words and creates word-concrete representation mappings through cross-situational learning, 3) Supervised grounding component (Section 3.3), which uses an interactive feedback based learning mechanism to improve the accuracy of word-concrete representation mappings as well as the acquisition speed. The unsupervised grounding component is based on an unsupervised grounding framework (Roesler, 2020b) that has recently been shown to outperform probabilistic model based approaches, which have been used in many previous grounding studies, e.g., (Tellex et al., 2011; Dawson et al., 2013; Aly et al., 2017; Roesler et al., 2019). The individual parts of the *hybrid* grounding framework are illustrated below and described in the following subsections.

1. **Percepts clustering**:
  • **Input**: Shape, color and, preposition percepts.
  • **Output**: Concrete representations of percepts.
2. **Cross-situational learning**:
  • **Input**: Natural language instructions, concrete representations of percepts, previously detected auxiliary words, and occurrence information of words and concrete representations.
  • **Output**: Set of auxiliary words and word to concrete representation mappings.
3. **Interactive learning**:
  • **Input**: Natural language instructions, concrete representations of percepts, set of auxiliary words and feedback information.
  • **Output**: Word to concrete representations mappings.

### 3.1 Percepts Clustering Component

The grounding mechanisms employed by the proposed framework (Sections 3.2 and 3.3) require that percepts are converted to concrete representations, which can be obtained through any clustering or classification algorithm. In this study, clustering is used because it neither requires labeled data nor explicit training, in comparison to classification algorithms. The employed clustering algorithm is DBSCAN^9^, which is a density-based clustering algorithm (Ester et al., 1996) that does not require the number of clusters to be known in advance, which is important because it is impossible to know in advance how many different shapes, colors, actions, or prepositions an agent will encounter when employed in the real world. The cluster numbers determined by DBSCAN are then provided to the grounding mechanisms to ground words through concrete representations by mapping words to cluster numbers, where the latter can be used to identify matching percepts.

### 3.2 Cross-Situational Learning Component

The cross-situational learning based grounding component is based on the unsupervised grounding framework proposed by Roesler (2020b) and uses cross-situational learning to create mappings between non-auxiliary words^10^ and their corresponding concrete representations. The approach proposed in Roesler (2020b) has been chosen for the unsupervised grounding component because it was able to ground actions, shapes, and colors more accurately and faster than probabilistic model based approaches, while also being able to successfully handle synonyms. However, two main changes have been made to the original framework that lead to slightly better auxiliary word detection and grounding results. First, the unsupervised grounding component does not have any restriction that all concrete representations need to be used once for grounding before any concrete representation can be used to ground multiple words. Second, the auxiliary word detection mechanism has been modified so that words only need to occur one time more than any concrete representation to be marked as an auxiliary word, however, they also need to occur at least two times. Together both changes lead to a slight increase in grounding performance as described in Section 5.1. Following the basic steps, illustrated through Algorithms 1, 2 are described.

Algorithm 1 The grounding procedure takes as input all words (*W*) and concrete representations (*CR*) of the current situation, the sets of all previously obtained word-concrete representation (*WCRPS*) and concrete representation-word (*CRWPS*) pairs, the set of auxiliary words (*AW*), and the set of permanent phrases (*PP*) and returns the sets of grounded words (*GW*) and grounded concrete representations (*GCR*).

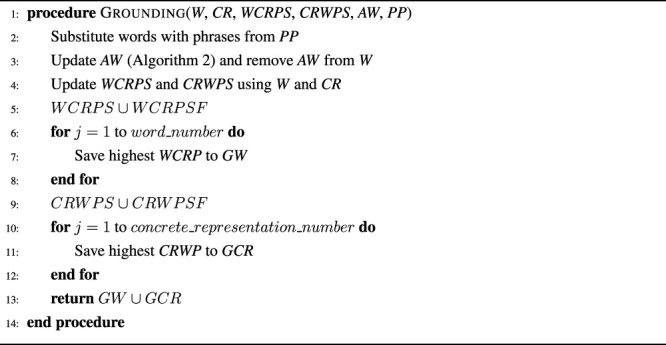

Algorithm 2 The auxiliary word detection procedure takes as input the sets of word and concrete representation occurrences (*WO* and *CRO*), and the set of all previously detected auxiliary words (*AW*).

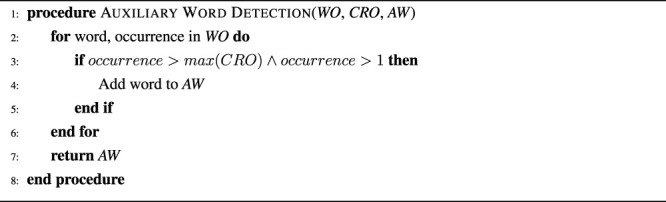

First, words that belong to a phrase are substituted by the phrase using a dictionary based approach, i.e. by checking whether any possible word sequence is part of the set of permanent phrases (*PP*). Theoretically, phrases can be automatically added to *PP* using machine learning, however, not much work exists on detecting phrases similar to the ones used in this study, e.g. “on the left side of” or “on the left of”, since they are only phrases in specific contexts and the latter of the examples is also part of the former^11^. Afterwards, auxiliary words are detected and removed from the current sentence by comparing word and concrete representation occurrences to identify words that occurred much more than any concrete representation (Algorithm 2). The sets of remaining words and concrete representations are then used to update the sets of word-concrete representation and concrete representation-word pairs (*WCRPS* and *CRWPS*). If feedback was provided during earlier situations, the feedback mappings (*WCRPSF* and *CRWPSF*) obtained by the interactive learning component (Section 3.3) are merged with *WCRPS* and *CRWPS*. Finally, the highest word-concrete representation and concrete representation-word pairs are determined for each word and concrete representation, respectively, and saved to the sets of words and concrete representations (*GW* and *GCR*).

### 3.3 Interactive Learning Component

The supervised or interactive learning component is inspired by the Grounded Naming Game methodology (Steels and Loetzsch, 2012), but has been designed so that it smoothly integrates with the unsupervised grounding component described in the previous section (Section 3.2). The main idea is to allow agents to receive and utilize non-verbal and verbal feedback from a tutor, when available, without depending on it. Feedback can consist of two parts: 1) pointing to the correct object, which allows the agent to identify the concrete representations belonging to the target object, and 2) an utterance, which provides a short description of the characteristics of the target object. While the first part, i.e., pointing to the correct object, is required for the interactive learning component to work, the second part, i.e. the utterance, is optional. The feedback is used by the agent to update its mappings to increase the probability that it identifies the target object correctly in similar situations in the future. Algorithm 3 provides an illustration of the two proposed feedback mechanisms. First, the set of non-target object concrete representations (*NOCR*) is calculated by subtracting the set of target object concrete representations (*TOCR*) from the set of all object concrete representations (*AOCR*). Afterwards, word-concrete representation and concrete representation-word feedback pairs are created or updated for each word in the instruction sentence and concrete representations in *TOCR*, if no verbal feedback is available. Otherwise, i.e., if verbal feedback is provided, feedback pairs are created or updated using the feedback sentence and each concrete representation in *TOCR* and *NOCR*. Thus, the feedback mechanism automatically takes into account verbal feedback (*WF*), if available, but does not require it because otherwise the instruction words (*WI*) will be used instead. The feedback mechanism has one parameter, i.e., *FRC*, which represents the feedback related change and determines how strong the influence of feedback is on the obtained mappings. *FRC* was initially set to two to ensure that feedback is twice as important as co-occurrence information, while ensuring that wrong feedback would not have a too strong influence. This setting was later also experimentally verified as the best setting. Feedback is integrated with the unsupervised algorithm by merging *WCRPS* and *WCRPSF* as well as *CRWPS* and *CRWPSF* in lines 5 and 9 of Algorithm 1 so that pairs that receive positive feedback are strengthened and pairs that receive negative feedback are weakened.

Algorithm 3 The feedback procedure takes as input the words of the instruction of the current situation (*WI*) and the feedback sentence (*WF*), the set of all object concrete representations (*AOCR*), the set of the target object concrete representations (*TOCR*), the set of detected auxiliary words (*AW*), and the sets of previously obtained word-concrete representation feedback (*WCRPSF*) and concrete representation-word feedback (*CRWPSF*) pairs, and returns updated *WCRPSF* and *CRWPSF*. The strength of the feedback is regulated through the parameter *FRC*, which was set to 2 for this study.

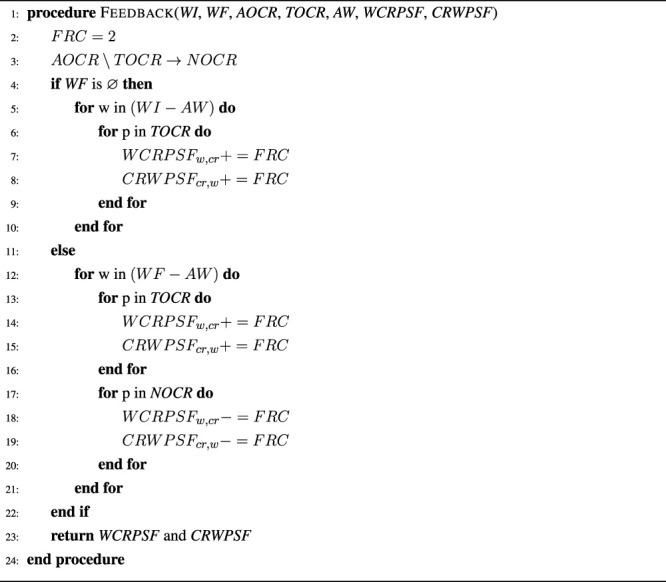

## 4 Experimental Setup

**FIGURE 1:**
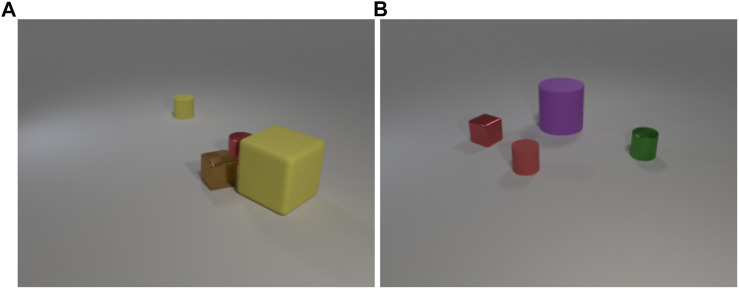
Two example scenes illustrating the used shapes and colors as well as the variation in size, material and light conditions. The corresponding sentences are: **(A)** “the red cylinder in front of the yellowish cylinder” and **(B)** “the red quadrate on the left side of the reddish cylinder”.

The 1,000 situations used in the experimental scenario are simulated using an environment based on the CLEVR dataset (Johnson et al., 2017). Every situation in the simulated environment consists of three or four objects with randomly chosen shapes, colors, materials, sizes and positions (Figure 1). Additionally, every situation has different light conditions, which adds noise to the perceived color information so that the similarity of two percepts of the same color varies depending on the light conditions. Three different modalities are extracted for each situation: 1) object shapes, which are represented by Viewpoint Feature Histogram (VFH) (Rusu et al., 2010) descriptors that encode the objects’ geometries and viewpoints, 2) object colors, which are represented by the mean RGB values of all object pixels, 3) preposition percepts, which are represented by 3D spatial vectors describing the spatial relation of the centroids of two objects. After all perceptual information have been obtained, a random sentence describing the generated scene is created, which has the following structure: “the *color shape preposition* the *color shape*,” where *color*, *shape*, and *preposition* are substituted by one of 12 shape, 16 color, and 6 preposition words/phrases (Table 1) to match the randomly selected target and reference objects. Most of the percepts can be referred to by several synonymous words to investigate how well the proposed framework handles synonymous words and phrases^12^. Additionally, each preposition word can be grounded through two homonymous prepositions, e.g., “on the right of” can be grounded through concrete representations 12 and 13, and “behind” can be grounded through concrete representations 12 and 14 (Table 1). The reason is that prepositions are not discrete because most objects need to be moved in two dimensions to reach the position of another object, therefore, if an object is in front of or behind another object it is most of the time also on the left or right of that object. Figure 1 illustrates this nicely because the red cylinder in Figure 1A is not just in front of the yellowish cylinder as indicated by the corresponding sentence, but also on the right side of it. Similarly the red quadrate in Figure 1B is both on the left side and behind of the reddish cylinder. Thus, two different concrete representations can be used to ground each of the preposition words.

**TABLE 1:** Overview of all concepts with their corresponding synonyms and concrete representation numbers (CR#) according to Figure 6.

Type	Concept	Synonyms	CR#
Shape	Cube	cube, block, hexahedron, quadrate	1
	Sphere	sphere, ball, spheroid, pellet, globe, orb, globule	2
	Cylinder	cylinder	3
Color	Gray	gray, grayish	4
	Red	red, reddish	5
	Blue	blue, blueish	6
	Green	green, greenish	7
	Brown	brown, brownish	8
	Purple	purple, purplish	9
	Cyan	cyan, greenish-blue	10
	Yellow	yellow, yellowish	11
Preposition	Right	on the right of, on the right side of	12, 13
	Front	in front of	13, 15
	Behind	behind	12, 14
	Left	on the left of, on the left side of	14, 15
Auxiliary Word	-	please	0

The obtained situations are then used to simulate human-agent interactions during which the human tutor asks the agent to select an object based on a natural language description. The employed interaction procedure is described below.

1. The human places three or four objects in front of the agent and the agent obtains the corresponding shape, color and preposition percepts and converts them to corresponding concrete representations.
2. The human provides a natural language description of the target object, e.g., “the red cylinder in front of the yellowish cylinder”.
3. The agent updates its word-concrete representation mappings using cross-situational learning (Section 3.2).
4. The agent identifies the target object and points to it.
5. If the agent pointed to the correct object, the human signals success, otherwise failure. Success or failure is either indicated through pointing and a verbal description, e.g. “yes the red cylinder” or “no the red cylinder,” or pointing-only, i.e., the human only points to the correct object which implicitly tells the agent whether it had selected the correct object.
6. The agent updates its sets of word-concrete representation and concrete representation-word feedback pairs based on the received feedback (Section 3.3).

The fifth and sixth steps are not necessary for the agent to learn the correct mappings, since it is, based on the obtained results (Section 5), able to learn them in an unsupervised manner. However, the feedback provided in step five is necessary for the supervised learning mechanism employed by the proposed framework (Section 3.3).

## 5 Results and Discussion

In this section the proposed hybrid continuous grounding framework is evaluated through two different human-agent interaction scenarios that differ in terms of the employed natural language utterances, perceptual features, and interaction procedures. The situations used in the first scenario are on the one hand rather simple because each situation contains only a singe object and the sentences are relatively short, while on the other hand every concrete representation can be referred to by at least two synonymous words and the used percepts have been obtained in a real environment. Additionally, the scenario has been used in previous grounding studies (Roesler et al., 2019; Roesler, 2020b) to evaluate unsupervised grounding approaches including an earlier version of the unsupervised grounding mechanism used by the proposed framework so that it provides a good opportunity to evaluate the latter. The second scenario consists of more situations with more complex natural language utterances and a larger number of concrete representations. Like the first scenario, the second scenario contains many synonyms but also homonyms, i.e., one word or phrase can be grounded through two or more concrete representations. The higher complexity of the second scenario is important because it is only possible to evaluate the benefit of combining unsupervised and supervised grounding approaches, if the groundings they obtain individually are not optimal. Thus, the second scenario is used to evaluate two different types of feedback and different feedback rates (Section 4) for the hybrid grounding framework.

### 5.1 Evaluation of the Unsupervised Grounding Component

**FIGURE 2:**
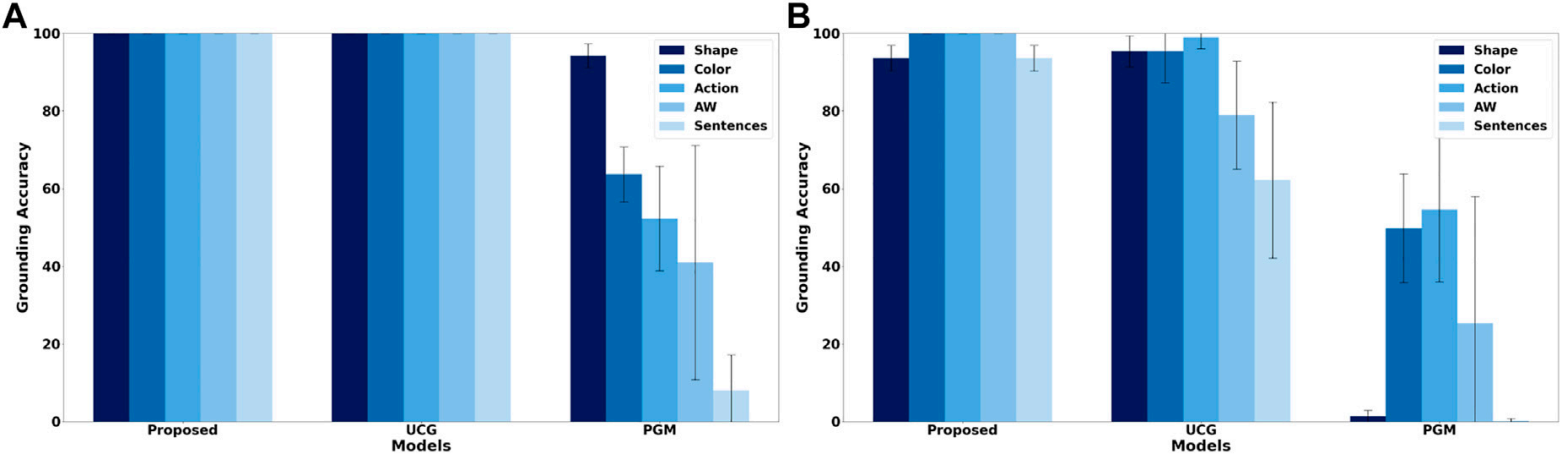
Mean grounding accuracy results, corresponding standard deviations, and percentage of sentences for which all words were correctly grounded for the probabilistic graphical model (PGM) and unsupervised continuous grounding framework (UCG) presented in (Roesler, 2020b) as well as the unsupervised grounding component of the hybrid grounding framework proposed in this study (Proposed) when employing them in the scenario described in (Roesler, 2020b). **(A)** shows the results when all situations are used for training and testing and **(B)** when 60% of the situations are used for training and the remaining 40% for testing.

**FIGURE 3:**
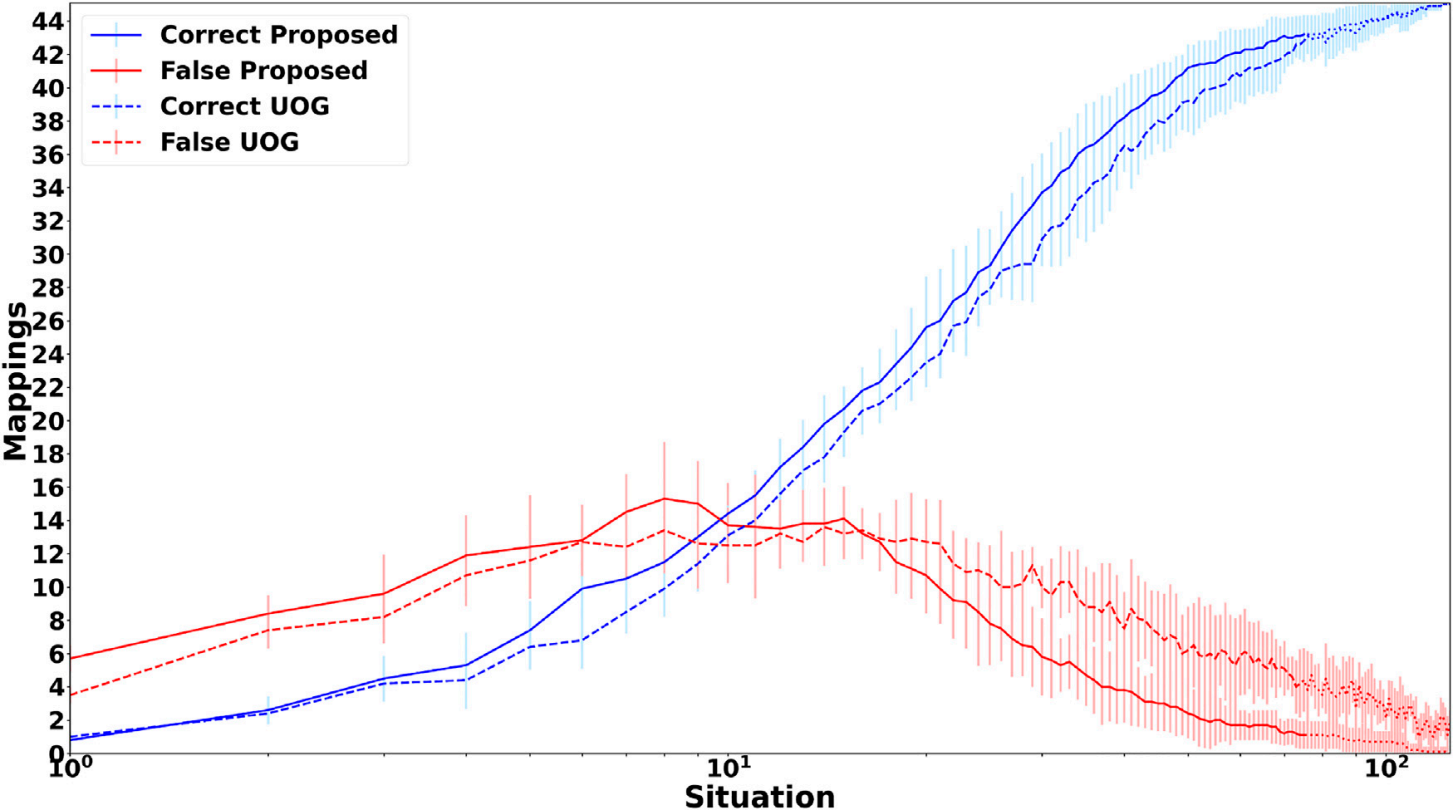
Mean number and standard deviation of correct and false mappings over all 125 situations of the scenario used in (Roesler, 2020b) for theunsupervised grounding component of the proposed hybrid grounding framework (Proposed) and the unsupervised continuous grounding framework (UCG) proposed in (Roesler, 2020b). The dotted part only occurs, when all situations are used for training.

Since the foundation of the proposed framework is a cross-situational learning based unsupervised grounding mechanism to ensure that it works when no feedback is provided, it is important to compare the groundings achieved by the unsupervised grounding component with other previously proposed unsupervised grounding models. To do this, the scenario used by Roesler (2020b),^13^ is employed, which consists of 125 situations described by shape, color and action percepts as well as an instruction with the following structure: “(please) *action* the *color shape*”, where *action*, *color*, and *shape* are replaced by one of 45 different words, while the auxiliary word “please” only appears in 44.8% of the situations. Five different shapes, colors and actions are included and each color and action can be referred to by two synonymous words, while shapes have five corresponding synonyms. Roesler (2020b) compared an earlier version of the unsupervised continuous grounding component (UCG) to a probabilistic graphical model (PGM), thereby, providing an easy way to compare the unsupervised grounding component to existing state-of-the-art grounding models. Figure 2 shows the accuracy of the obtained groundings for all three models and two different cases. For the first case (Figure 2A) all situations are encountered during training and testing because both the proposed framework and UCG are able to learn continuously so that no separate offline training phase is required. However, this case is unrealistic for PGM because it requires an offline training phase and it is very unlikely that it encounters all possible situations already during training. Thus, in the second case (Figure 2B) only 60% of the situations are used for training, thus, the learning mechanisms of the proposed framework and UCG are deactivated after 60% of the situations have been encountered. Figure 2 shows that for the first case the continuous learning frameworks are able to achieve perfect groundings. In contrast, PGM only achieves more than 90% accuracy for shapes, while for actions and auxiliary words it achieves only accuracies around or below 50%. For the second case, which is more realistic for PGM while introducing an unrealistic restriction for the continuous grounding frameworks, the grounding accuracies for all frameworks drop. For PGM the largest decrease is for shapes. This large decrease might be due to the higher complexity of the shape percepts (308 dimensional vectors) in comparison to action (30 dimensional vectors) and color (10 dimensional vectors) percepts so that the lower number of training situations is not sufficient for the baseline model to learn the correct groundings. For the continuous grounding frameworks (Proposed and UCG) the accuracies decrease much less, nevertheless the results show that the changes described in Section 3.2 improve the auxiliary word detection algorithm so that the revised algorithm is able to detect all auxiliary words for the employed scenario. Additionally, the change to the cross-situational learning mechanism, i.e., removing the restriction that all concrete representations need to be used once for grounding before a concrete representation can be used to ground multiple words, improves both the accuracy of the obtained action groundings (Figure 2B) as well as the speed of convergence towards the correct mappings (Figure 3)^14^, while it causes only a slight decrease of the grounding accuracy for shapes. Overall, the results illustrate the large accuracy improvement when comparing the proposed unsupervised grounding algorithm (Proposed) with a state-of-the-art probabilistic model (PGM), which is similar to other unsupervised learning models that have been used in many previous grounding studies, like (Tellex et al., 2011; Dawson et al., 2013; Aly et al., 2017; Roesler et al., 2019). Besides the better grounding accuracy, the proposed algorithm has the additional advantage that it does not require an explicit training phase but continuously integrates new words and concrete representations into its set of word-concrete representation mappings as illustrated by Figure 3, which makes it more applicable for real world human-agent interactions because it is impossible to create a large enough training set to cover all possible situations that could occur during these interactions.

### 5.2 Evaluation of the Hybrid Grounding Framework

The hybrid grounding framework is evaluated through a simulated human-agent interaction scenario (Section 4). The main questions investigated in this section are whether combining unsupervised and supervised grounding approaches leads to more sample efficient, accurate and flexible grounding than using only one of the two paradigms and whether combined pointing and verbal feedback provides a benefit over pointing-only feedback.

**FIGURE 4:**
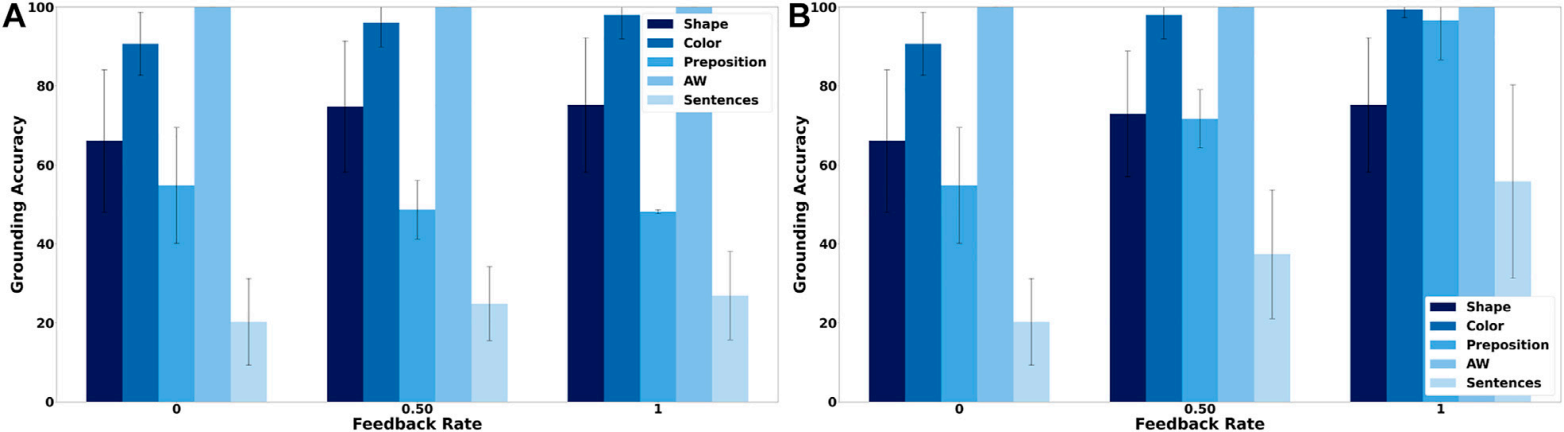
Mean grounding accuracy results, corresponding standard deviations, and percentage of sentences for which all words were correctly grounded for both types of feedback. **(A)** shows the grounding results when pointing-only feedback is provided and **(B)** when both pointing and verbal feedback is provided.

**FIGURE 5:**
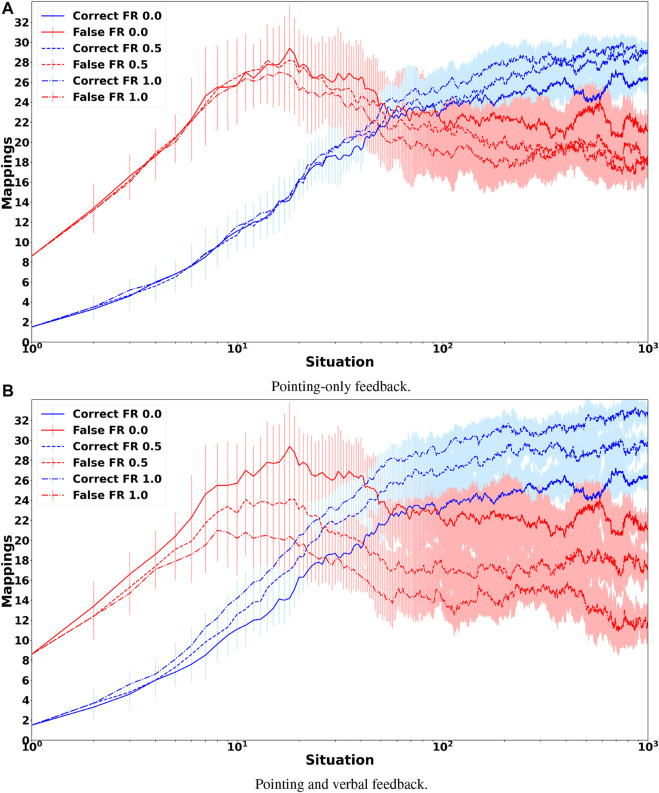
Mean number and standard deviation of correct and false mappings over all 1,000 situations, when feedback is provided for 0%, 50% or 100% of the situations, where FR means feedback rate. **(A)** shows the results when pointing-only feedback is provided and **(B)** when pointing and verbal feedback is provided.

**FIGURE 6:**
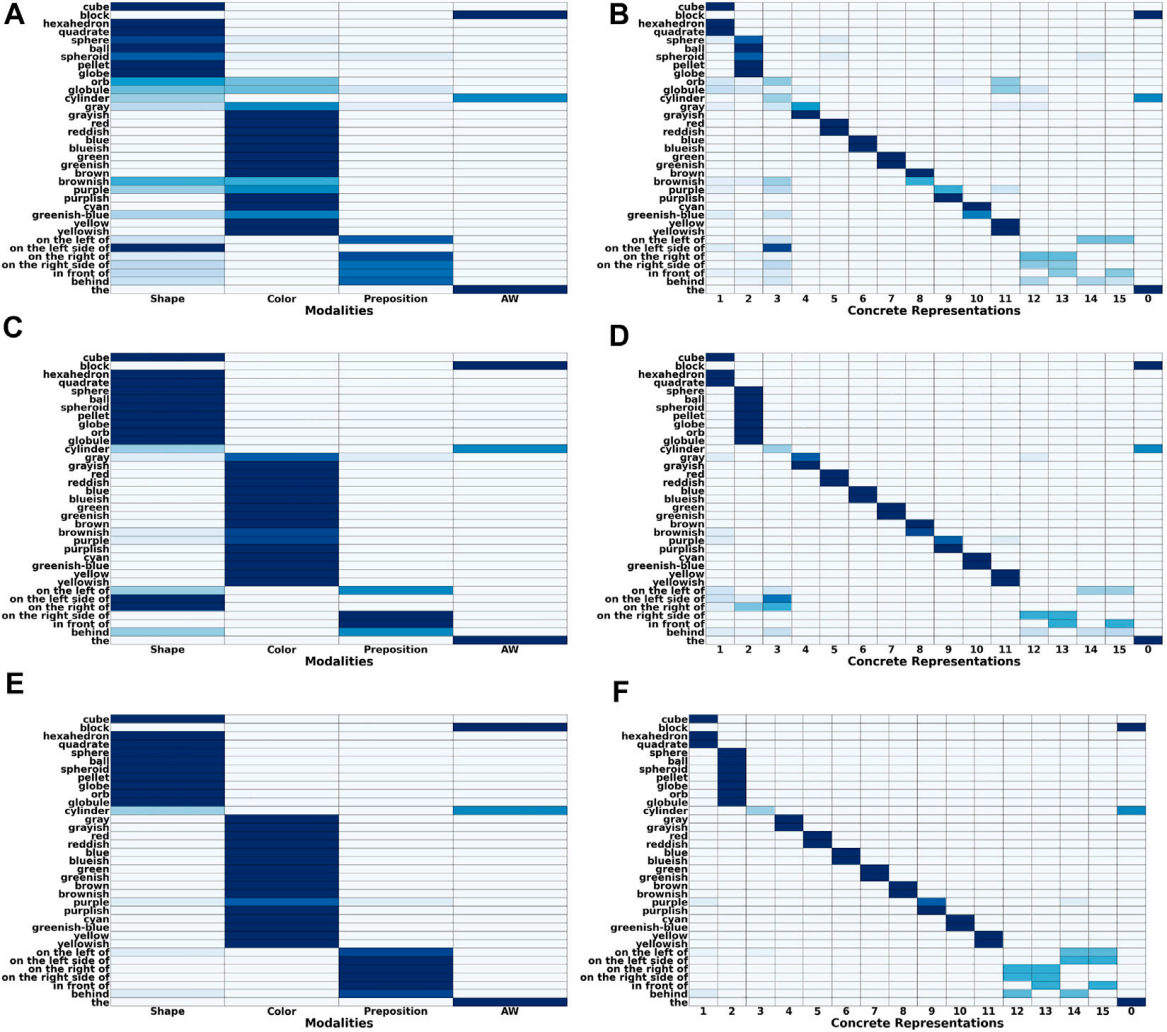
Confusion matrices of words over different modalities (left side) and words over different concrete representations (right side) for all ten situation sequences and three different types of interactions, i.e., no feedback **(A, B)**, pointing-only feedback **(C, D)** and combined verbal and pointing feedback **(E, F)**.

Figure 4A shows the grounding results when only non-verbal pointing-only feedback is provided. It clearly shows that the feedback mechanism has only a small mostly positive effect on the accuracy of the obtained groundings. In comparison, when the tutor also provides verbal feedback the accuracy of the obtained groundings improves visibly (Figure 4B) with the number of correctly grounded sentences increasing from about 20% to more than 50%. This increase is mostly due to an increase in the grounding accuracy of prepositions as well as colors, while the accuracy of shape groundings increases only slightly. The reason for the latter is that the lower accuracy for shapes is mostly due to the words “block” and “cylinder” being incorrectly classified as auxiliary words so that the availability of feedback only provides limited benefit because it has no influence on the auxiliary word detection algorithm. Figure 5 shows how the number of correct and false mappings changes over all 1,000 situations for different feedback rates, i.e. depending on how often feedback is given. For pointing-only feedback the final number of correct mappings, i.e., after 1,000 situations, increases when feedback is provided for at least 50% of the situations, while the final accuracy does not increase further, if feedback is provided for more situations. However, when feedback is provided for all situations, the number of correct mappings increases faster than when feedback is only provided for 50% of the situations (Figure 5A). For combined pointing and verbal feedback there is a clear difference regarding the final grounding accuracy as well as the speed correct mappings are obtained (Figure 5B). For example, when no feedback is provided, it takes more than 90 situations until the number of correct mappings is equal to the number of false mappings, while it only takes about 27 and 19 situations when feedback is given for 50% of the situations or all situations, respectively. Additionally, after all 1,000 situations have been encountered the number of correct mappings is about 14% higher if feedback is provided on average every second situation than if no feedback is provided, followed by another 11% increase, if feedback is provided every situation. These results illustrate the benefit of verbal feedback in addition to pointing feedback. However, the results also show that the framework does not depend on feedback and achieves decent grounding results, if no feedback is provided, which is important because the availability of feedback cannot be guaranteed. Figures 3, 5 also illustrate the continuous learning ability of the proposed framework, which is very important when considering deployment in real environments that require open-ended learning because it is impossible to create a large enough dataset that contains all possible words and concrete representations that an agent could encounter. In addition, it also shows the transparency and explainability of the framework because at any time it is possible to check the current mappings and understand why they have been created based on the available co-occurrence information stored in WCRPS, CRWPS, WCRPSF, and CRWPSF (Sections 3.2 and 3.3).While the accuracies provide a good overview of how accurately the groundings are for each modality, they neither provide any details about the accuracy of the groundings obtained for individual words nor any details about the wrong mappings. Therefore, Figure 6A shows the confusion matrix for all words and modalities, which illustrates how often each word was grounded through the different modalities, when no feedback is provided. The figure shows that there is some confusion between shapes and colors as well as prepositions and shapes, while overall most words are grounded through the correct modality. When pointing-only feedback is provided for every situation the confusion for shapes disappears and the confusion for colors also decreases (Figure 6C). For prepositions the change is bidirectional, i.e., for two prepositions the confusion gets less while for two other prepositions they are more often grounded through shapes. However, this confusion disappears when combined verbal and pointing feedback is provided every situation (Figure 6E). In that case, there is only very light confusion for “purple,” “on the left of,” and “behind”. Since grounding is not about determining the modality a word belongs to but to create a mapping from words to corresponding concrete representations, it is important to also look at the confusion matrices of words over different concrete representations. Figure 6B shows the confusion matrix of words over different concrete representations when no feedback is provided. The figure shows that there is not much intra-modality confusion and that most of the inter-modality confusion is for the concrete representations of the shapes, i.e., concrete representations 1, 2 and 3, and yellow, i.e., concrete representation 11, because many words are incorrectly mapped to them, although all mappings except for “on the left side of” are relatively weak. For the preposition words it is interesting to see that most of them are mapped to two concrete representations, which is correct because all prepositions should be grounded through two homonymous concrete representations. When looking at Figure 6C, which shows the confusion matrix for the case were pointing-only feedback is provided for every situation, it is interesting to see that the mappings for prepositions are less accurate and weaker. The reason for this is that pointing-only feedback strengthens the mappings from the concrete representations of the target object’s shape and color with all words of the utterance. Thus, the mappings from the preposition words to the concrete representations of shape and color are strengthened as well, the former even more because there are only three different concrete representations for shapes in comparison to eight for colors. However, when combined verbal and pointing feedback is provided the confusion for prepositions is gone and in general there is nearly no confusion (Figure 6F). The former is due to the availability of the feedback sentence which ensures that only the mappings from the color and shape words of the target object to the corresponding concrete representations are strengthened. This clearly shows the importance of verbal feedback when comparing it to the pointing-only feedback case, while the confusion matrices also showed that the proposed framework is also able to achieve decent groundings, if no feedback is available, which confirms the results presented in Section 5.1.

## 6 Conclusions and Future Work

This paper investigated whether combining unsupervised and supervised grounding mechanisms improves the sample-efficiency and accuracy of the former, while avoiding the latter to fail in the absence of supervision. More specifically, a hybrid grounding framework, which uses cross-situational learning to ground words in an unsupervised manner, while being able to utilize pointing-only or combined pointing and verbal feedback to speed up the grounding process and improve the accuracy of the obtained groundings, was evaluated through a simulated human-agent interaction scenario. The results showed that the ability to learn from human feedback improves both the sample-efficiency and accuracy of the framework. When only non-verbal feedback in form of pointing is provided the improvement is only minor and only for concrete representation that can be pointed at, e.g., shapes or colors, while it has a negative effect on concrete representations of concepts that cannot be pointed at, e.g., prepositions. In comparison, when also verbal feedback is provided, the grounding accuracy improves substantially achieving nearly perfect grounding for colors and prepositions. Additionally, the results also showed that the proposed framework is still able to correctly detect auxiliary words and ground a large number of non-auxiliary words correctly when no feedback is provided, which is very important because it cannot be assumed that a tutor who is willing to provide feedback is always available. In future work, it will be investigated how robust the proposed feedback mechanism is regarding false feedback, e.g., the tutor points to the wrong object or uses a wrong word to describe the target object. Additionally, it will be investigated whether the framework can be extended to benefit from explicit teaching as described by Roesler (2020a). Finally, the framework will be evaluated for scenarios in which the agent has to manipulate the target object instead of just pointing to it.

## Data Availability

The raw data supporting the conclusion of this article will be made available by the author upon request.

## Footnotes

1
Harnad (1990) calls them “categorical representations” that are created by reducing “iconic representations” of inputs, which are obtained through an agent’s sensors, to a set of invariant features that are sufficient to distinguish percepts belonging to different concepts.

2 The meaning of the term *concept* is still an area of active philosophical debate (see e.g. (Margolis and Laurence, 2007; Zalta, 2021)) and in most grounding studies it is either used synonymous to words or symbols, e.g., Stramandinoli et al. (2011); Aly et al. (2017), or it is completely avoided by directly stating that words are grounded through concrete representations, e.g., (Nakamura et al., 2009; Marocco et al., 2010). Since the scenario employed in this study contains synonyms and homonyms, concepts can neither be represented through words nor concrete representations, instead they are implicitly represented by the connections between words and concrete representations.

3 The two investigated types of feedback, i.e., pointing-only feedback and combined verbal and pointing feedback are described in detail in Section 3.3.

4 A concept can be referred to by multiple words (synonyms), while one word can refer to multiple concepts (homonyms). One concept can then be grounded through multiple concrete representations (homonyms), while it is not possible to have synonymous concepts, i.e. multiple concepts being grounded through the same concrete representation. Since the framework employed in this study represents concepts only implicitly, synonyms are words that refer to the same concrete representation and homonyms are concrete representations that refer to the same word.

5 The “Principle of Contrast” states that no two words refer to the exact same meaning, i.e. there are no true synonyms, but words can be synonyms in specific contexts (Clark, 1987). For example, *chocolate* and *sweets* are usually not synonymous because *sweets* has a broader meaning, however, when there is only one box of chocolate on the table and someone asks for the *chocolate* or *sweets* the words are synonymous in that context because they have same meaning, i.e., they refer to the same object.

6 Word2Vec uses a large corpus of plain text as input and outputs a vector space, where each distinct word is represented by a vector and the distance between two vectors corresponds to the syntactic-semantic similarity between two corresponding words Mikolov et al. (2013a,b).

7 For example, to teach the artificial agent the higher level symbol “grab,” the tutor would instruct the agent to first “open” its gripper, “move” to the target object, and then “close” its gripper, relying on “open,” “move,” and “close” to be already grounded.

8 Even the utterances used to name novel objects in word learning studies with young children or infants are complete grammatically correct sentences, like “Look, this is the cheem!” (Horst and Samuelson, 2010), and not just single words, like “Cheem!”.

9 The employed version of DBSCAN is available in *scikit-learn* (Pedregosa et al., 2011).

10 Auxiliary words are words that only exist for grammatical reasons, e.g., “the” or “while”, and do not have corresponding concrete representations.

11
Roesler and Nowé (2019) proposed a cross-situational learning based approach to automatically detect phrases very similar to the ones employed in this study, however, the mechanism did not work reliably for the sentences in this study and has therefore not been used.

12 For example, the concept **cube** can be referred to by the words *cube*, *block*, *hexahedron*, and *quadrate*, while the concept **red** can be referred to by the words *red* and *reddish* (Table 1).

13 The dataset is currently not publicly available, but can be obtained from the author on request.

14 Due to the need for an offline training phase, no corresponding results can be obtained for PGM.

## References

[B1] Akhtar N. Montague L. (1999). Early Lexical Acquisition: the Role of Cross-Situational Learning. First Lang. 19, 347–358. 10.1177/014272379901905703

[B2] Aly A. Taniguchi A. Taniguchi T. (2017). “A Generative Framework for Multimodal Learning of Spatial Concepts and Object Categories: An Unsupervised Part-Of-Speech Tagging and 3D Visual Perception Based Approach,” in IEEE International Conference on Development and Learning and the International Conference on Epigenetic Robotics (ICDL-EpiRob), Lisbon, Portugal. 10.1109/devlrn.2017.8329833

[B3] American Psychiatric Association and others (2013). Diagnostic and Statistical Manual of Mental Disorders (DSM-5®). 5th edition. Washington, DC: American Psychiatric Pub.

[B4] Bedford R. Gliga T. Frame K. Hudry K. Chandler S. Johnson M. H. (2013). Failure to Learn from Feedback Underlies Word Learning Difficulties in Toddlers at Risk for Autism. J. Child. Lang. 40, 29–46. 10.1017/S0305000912000086 23217290 PMC3518974

[B5] Belpaeme T. Morse A. (2012). Word and Category Learning in a Continuous Semantic Domain: Comparing Cross-Situational and Interactive Learning. Adv. Complex Syst. 15. 10.1142/s0219525912500312

[B6] Bleys J. Loetzsch M. Spranger M. Steels L. (2009). “The Grounded Color Naming Game,” in Proceedings of the 18th IEEE International Symposium on Robot and Human Interactive Communication (RO-MAN).

[B7] Bloom P. (2001). Précis of How Children Learn the Meanings of Words. Behav. Brain Sci. 24, 1095–1103. 10.1017/s0140525x01000139 12412326

[B8] Blythe R. A. Smith K. Smith A. D. M. (2010). Learning Times for Large Lexicons through Cross-Situational Learning. Cognitive Sci. 34, 620–642. 10.1111/j.1551-6709.2009.01089.x 21564227

[B9] Clark E. V. (1987). “The Principle of Contrast: A Constraint on Language Acquisition,” in Mechanisms of Language Acquisition (Mahwah, NJ: Lawrence Erlbaum Associates), 1–33.

[B10] Dawson C. R. Wright J. Rebguns A. Escárcega M. V. Fried D. Cohen P. R. (2013). “A Generative Probabilistic Framework for Learning Spatial Language,” in IEEE Third Joint International Conference on Development and Learning and Epigenetic Robotics (ICDL), Osaka, Japan. 10.1109/devlrn.2013.6652560

[B11] Ellis D. G. (1993). Language and Communication. Commun. Educ. 42, 79–92. 10.1080/03634529309378914

[B12] Ester M. Kriegel H.-P. Sander J. Xu X. (1996). “A Density-Based Algorithm for Discovering Clusters in Large Spatial Databases with Noise,” in Proceedings of the 2nd International Conference on Knowledge Discovery and Data Mining (KDD), Portland, Oregon, USA, 226–231.

[B13] Fisher C. Hall D. G. Rakowitz S. Gleitman L. (1994). When it Is Better to Receive Than to Give: Syntactic and Conceptual Constraints on Vocabulary Growth. Lingua 92, 333–375. 10.1016/0024-3841(94)90346-8

[B14] Fontanari J. F. Tikhanoff V. Cangelosi A. Ilin R. Perlovsky L. I. (2009). Cross-situational Learning of Object-word Mapping Using Neural Modeling Fields. Neural Netw. 22, 579–585. 10.1016/j.neunet.2009.06.010 19596549

[B15] Harnad S. (1990). The Symbol Grounding Problem. Phys. D. Nonlinear Phenom. 42, 335–346. 10.1016/0167-2789(90)90087-6

[B16] Horst J. S. Samuelson L. K. (2010). Fast Mapping but Poor Retention by 24-Month-Old Infants. Infancy 13, 128–157. 10.1080/1525O00070179559 33412722

[B17] Johnson J. Hariharan B. van der Maaten L. Fei-Fei L. Zitnick C. L. Girshick R. (2017). “Clevr: A Diagnostic Dataset for Compositional Language and Elementary Visual Reasoning,” in Proceedings of the IEEE Conference on Computer Vision and Pattern Recognition (CVPR), 2901–2910. 10.1109/cvpr.2017.215

[B18] Margolis E. Laurence S. (2007). The Ontology of Concepts-Abstract Objects or Mental Representations? Nous 41, 561–593. 10.1111/j.1468-0068.2007.00663.x

[B19] Marocco D. Cangelosi A. Fischer K. Belpaeme T. (2010). Grounding Action Words in the Sensorimotor Interaction with the World: Experiments with a Simulated Icub Humanoid Robot. Front. Neurorobot 4. 10.3389/fnbot.2010.00007 PMC2901088 20725503

[B20] Mikolov T. Chen K. Corrado G. Dean J. (2013a). Efficient Estimation of Word Representations in Vector Space. ArXiv e-prints. Eprint: 1301.3781.

[B21] Mikolov T. Yih t. W. Zweig G. (2013b). “Linguistic Regularities in Continuous Space Word Representations,” in Proceedings of the 2013 Conference of the North American Chapter of the Association for Computational Linguistics: Human Language Technologies (NAACL-HLT-2013) (Stroudsburg, Pennsylvania, USA: Association for Computational Linguistics).

[B22] Nakamura T. Nagai T. Iwahashi N. (2009). “Grounding of Word Meanings in Multimodal Concepts Using LDA,” in Proceedings of the 2009 IEEE/RSJ International Conference on Intelligent Robots and Systems (IROS). 10.1109/iros.2009.5354736

[B23] Nevens J. Spranger M. (2017). “Computational Models of Tutor Feedback in Language Acquisition,” in 7th Joint IEEE International Conference on Development and Learning and on Epigenetic Robotics (ICDL-EpiRob), Lisbon, Portugal. 10.1109/devlrn.2017.8329811

[B24] Pedregosa F. Varoquaux G. Gramfort A. Michel V. Thirion B. Grisel O. (2011). Scikit-learn: Machine Learning in python. J. Mach. Learn. Res. 12, 2825–2830.

[B25] Pinker S. (1989). Learnability and Cognition. Cambridge, MA: MIT Press.

[B26] Roesler O. Aly A. Taniguchi T. Hayashi Y. (2018). “A Probabilistic Framework for Comparing Syntactic and Semantic Grounding of Synonyms through Cross-Situational Learning,” in ICRA-18 Workshop on Representing a Complex World: Perception, Inference, and Learning for Joint Semantic, Geometric, and Physical Understanding, Brisbane, Australia.

[B27] Roesler O. Aly A. Taniguchi T. Hayashi Y. (2019). “Evaluation of Word Representations in Grounding Natural Language Instructions through Computational Human-Robot Interaction,” in Proceedings of the 14th ACM/IEEE International Conference on Human-Robot Interaction (HRI), Daegu, South Korea, 307–316. 10.1109/hri.2019.8673121

[B28] Roesler O. (2020a). “Enhancing Unsupervised Natural Language Grounding through Explicit Teaching,” in Proc. of the UKRAS20 Conference: “Robots into the real world”, Lincoln, UK. 10.31256/bf9vw8c

[B29] Roesler O. Nowé A. (2019). Action Learning and Grounding in Simulated Human Robot Interactions. Knowl. Eng. Rev. 34. 10.1017/s0269888919000079

[B30] Roesler O. (2020b). “Unsupervised Online Grounding of Natural Language during Human-Robot Interaction,” in Second Grand Challenge and Workshop on Multimodal Language at ACL 2020, Seattle, USA.

[B31] Rusu R. B. Bradski G. Thibaux R. Hsu J. (2010). “Fast 3D Recognition and Pose Using the Viewpoint Feature Histogram,” in Proceedings of the 2010 IEEE/RSJ International Conference on Intelligent Robots and Systems (IROS), Taipei, Taiwan, 2155–2162. 10.1109/iros.2010.5651280

[B32] She L. Yang S. Cheng Y. Jia Y. Chai J. Y. Xi N. (2014). “Back to the Blocks World: Learning New Actions through Situated Human-Robot Dialogue,” in Proceedings of the SIGDIAL 2014 Conference, Philadelphia, U.S.A., 89–97. 10.3115/v1/w14-4313

[B33] Siskind J. M. (1996). A Computational Study of Cross-Situational Techniques for Learning Word-To-Meaning Mappings. Cognition 61, 39–91. 10.1016/s0010-0277(96)00728-7 8990968

[B34] Smith A. D. M. Smith K. (2012). Cross-Situational Learning. Boston, MA: Springer US, 864–866. 10.1007/978-1-4419-1428-6_1712

[B35] Smith K. Smith A. D. M. Blythe R. A. (2011). Cross-situational Learning: An Experimental Study of Word-Learning Mechanisms. Cognitive Sci. 35, 480–498. 10.1111/j.1551-6709.2010.01158.x

[B36] Smith L. Yu C. (2008). Infants Rapidly Learn Word-Referent Mappings via Cross-Situational Statistics. Cognition 106, 1558–1568. 10.1016/j.cognition.2007.06.010 17692305 PMC2271000

[B37] Spranger M. (2013). “Grounded Lexicon Acquisition - Case Studies in Spatial Language,” in IEEE Third Joint International Conference on Development and Learning and Epigenetic Robotics (ICDL-Epirob), Osaka, Japan.

[B38] Steels L. Loetzsch M. (2012). “The Grounded Naming Game,” in Experiments in Cultural Language Evolution. Editor Steels L. (Amsterdam: John Benjamins), 41–59. 10.1075/ais.3.04ste

[B39] Stramandinoli F. Cangelosi A. Marocco D. (2011). “Towards the Grounding of Abstract Words: A Neural Network Model for Cognitive Robots,” in The 2011 International Joint Conference on Neural Networks, San Jose, CA, USA. 10.1109/ijcnn.2011.6033258

[B40] Tellex S. Kollar T. Dickerson S. Walter M. R. Banerjee A. G. Teller S. (2011). Approaching the Symbol Grounding Problem with Probabilistic Graphical Models. AIMag 32, 64–76. 10.1609/aimag.v32i4.2384

[B41] Zalta E. N. (2021). Unifying Three Notions of Concepts. Theoria 87, 13–30. 10.1111/theo.12187

